# Prenatal cannabinoid exposure alters excitation-inhibition balance through glutamate and GABA receptor-mediated signaling

**DOI:** 10.1016/j.jbc.2026.113196

**Published:** 2026-05-27

**Authors:** Priyanka D. Pinky, Warren D. Smith, Miles T. Wiley, Jenna Bloemer, Vasiliki Syropoulou, Iva Durdanovic, Sharay E. Setti, Jeremiah C. Pfitzer, Savannah R. Perman, Kawsar U. Chowdhury, Kelli L. McDonald, Michael W. Gramlich, Subhrajit Bhattacharya, Vishnu Suppiramaniam, Miranda N. Reed

**Affiliations:** 1 Department of Drug Discovery and Development, Harrison College of Pharmacy, Auburn University, Auburn, Alabama, USA; 2 Center for Neuroscience Initiative, Auburn University, Auburn, Alabama, USA; 3 Department of Pharmaceutical Sciences, Mercer University College of Pharmacy, Atlanta, Georgia, USA; 4 Department of Electrical and Computer Engineering, Herbert Wertheim College of Engineering, University of Florida, Gainesville, Florida, USA; 5 School of Pharmacy, Keck Graduate Institute, The Claremont Colleges, Claremont, California, USA; 6 Physics Department, Auburn University, Auburn, Alabama, USA; 7 Department of Molecular and Cellular Biology, College of Science and Mathematics, Kennesaw State University, Kennesaw, Georgia, USA

**Keywords:** prenatal, cannabinoid, memory, behavior, developmental, glutamate, synaptic plasticity, marijuana, adolescence, cannabis

## Abstract

The growing perception that marijuana is safe during pregnancy has led to a marked increase in prenatal cannabis use, raising concerns about its long-term effects on brain development and cognition. This study investigated the consequences of prenatal delta-9-tetrahydrocannabinol (THC) exposure on hippocampal circuit function, synaptic plasticity, and memory in adolescent offspring using a rodent model. We found that prenatal THC exposure resulted in persistent deficits in hippocampal-dependent memory and significant disruptions in synaptic plasticity, including impaired long-term potentiation and increased long-term depression. Electrophysiological analyses revealed reduced AMPAR-mediated synaptic transmission and a shift toward increased inhibitory signaling, suggesting an excitation/inhibition (E/I) imbalance in the hippocampus. These functional changes were accompanied by selective downregulation of postsynaptic glutamatergic proteins (GluA1, GluN2A, GluN2B, and PSD95), while presynaptic glutamate markers remained unchanged. Notably, immunohistochemical and anatomical analyses demonstrated region-specific reorganization of inhibitory networks, including altered distribution and colocalization of cannabinoid 1 receptor (CB_1_R) and vesicular GABA transporter (VGAT) across hippocampal subregions. Together, our results reveal that prenatal THC exposure leads to coordinated functional and structural remodeling of hippocampal circuits, producing a lasting E/I imbalance and memory impairments during adolescence. These findings highlight disrupted GABAergic signaling as a potential therapeutic target for mitigating cognitive deficits resulting from prenatal cannabis exposure.

In recent years, the perception that marijuana is safe for use during pregnancy has increased (1), leading to a dramatic increase in cannabis use during pregnancy (2, 3). This is particularly concerning given that cannabinoids readily cross the placental barrier to expose the fetus (4, 5). Despite the recent increase in studies examining the consequences of perinatal cannabis exposure (*e.g.*, (6, 7, 8, 9, 10, 11)), prenatal cannabis use has been relatively understudied compared to other drugs of abuse. The cannabinoid receptor type 1 (CB_1_R) is expressed early during prenatal development and is functionally coupled to stransduction mechanisms from early prenatal stages in both rodents (12) and humans (13). The cannabinoid system is critical in neurodevelopment, affecting synaptogenesis, proliferation, and migration of neuronal cells, functional synaptic organization, and signal transduction (as reviewed in (14)). Due to the high density of CB_1_Rs in brain regions implicated in higher cognitive function, including the hippocampus and prefrontal cortex (15, 16), persistent cognitive alterations are likely to occur. In humans, for example, prenatal cannabinoid exposure is associated with impairments in memory and attention during adolescence (17, 18). However, the potential mechanisms for these long-lasting cognitive changes remain unknown. One potential mechanism could be perturbation of glutamatergic neurotransmission (19), which plays a critical role in synaptic plasticity and memory formation, as shown in previous studies following cannabinoid exposure (20, 21). However, emerging evidence suggests that the underlying mechanisms of altered memory may also include an imbalance in glutamatergic and gamma-aminobutyric acid (GABA)-ergic signaling, the major excitatory and inhibitory neurotransmitters, respectively, in the brain (22). A balanced cooperation of these systems is needed for proper synaptic stability and synchronous firing in the hippocampus (23, 24), and their interplay in spontaneous synaptic communication is altered after delta-9-tetrahydrocannabinol (THC) exposure (25, 26, 27). Studies have also shown that CB_1_Rs induce short bursts of long-term depression (LTD) in hippocampal synapses that are known to impair memory formation (28, 29, 30). Collectively, prior work suggests that cannabinoids perturb excitatory and inhibitory signaling and CB_1_R-mediated plasticity, motivating investigation into whether prenatal cannabis exposure leads to persistent alterations in inhibitory circuitry during adolescence.

Although prenatal THC exposure has been linked to early life synaptic alterations (31), its effects on adolescent hippocampal circuit architecture and inhibitory network maturation have not been systematically examined. Adolescence represents a critical developmental window marked by extensive synaptic refinement and maturation of inhibitory networks (32), yet most prenatal THC studies have focused on early postnatal periods. Moreover, prior work has largely emphasized global changes in receptor expression or plasticity phenotypes (20) rather than examining whether prenatal THC exposure produces lasting presynaptic and anatomical reorganization of inhibitory circuitry at this later stage. Whether cannabinoids alter the distribution of inhibitory terminals across hippocampal networks or the colocalization of CB_1_Rs with GABAergic synapses during adolescent circuit maturation remains unknown. Addressing this gap is essential for linking early life exposure to persistent excitation–inhibition (E/I) imbalance and memory dysfunction.

In the current study, we used a rodent model of prenatal THC exposure to examine the long-lasting consequences on hippocampal glutamatergic neurotransmission and memory during adolescence. Prenatal THC exposure caused deficits in hippocampal basal synaptic transmission and synaptic plasticity that were associated with deficits in hippocampal-dependent memory during adolescence. These memory deficits were confirmed further with field recordings showing impaired long-term potentiation (LTP) and increased LTD, cellular substrates for memory formation. To further dissect the role of glutamate signaling in such deficits and to understand the role of E/I mediated by glutamatergic and GABAergic systems, we used patch clamp electrophysiology to examine alterations in spontaneous events and spike-frequency, measures of synaptic communication and activity threshold. Our data suggest that THC exposure impairs AMPAR-mediated synaptic transmission with an overall shift toward inhibitory currents. The alteration in glutamatergic signaling was further confirmed by microelectrode array (MEA) recordings suggesting increased quanta of release, which may have contributed to sustained desensitization of AMPAR signaling. Our immunoblotting data revealed selective reductions in postsynaptic glutamatergic proteins, including GluA1 and the NMDAR subunits GluN2A and GluN2B, as well as decreased expression of the postsynaptic scaffolding protein PSD95. In contrast, presynaptic vesicle markers VGLUT1 and synaptophysin were unchanged, indicating preserved presynaptic terminal structure despite functional alterations in release dynamics for animals exposed prenatally to THC. We next asked whether prenatal THC exposure produces lasting functional changes in the presynaptic organization of inhibitory circuits that could account for the observed shifts in synaptic recruitment and plasticity. In particular, we examined stimulation–response relationships for inhibitory and excitatory inputs and quantified the spatial distribution and colocalization of CB_1_R with the vesicular GABA transporter (VGAT) across hippocampal subregions. These analyses revealed region-specific remodeling of inhibitory circuitry, including altered inhibitory recruitment thresholds and redistribution of CB_1_R in hippocampal subregions, providing a structural framework for the E/I imbalance observed following prenatal THC exposure. Together, our findings indicate that prenatal THC exposure produces long-lasting E/I imbalance through coordinated functional and anatomical remodeling of hippocampal circuits, highlighting disrupted GABA signaling as a potential avenue for therapeutic intervention to restore memory deficits caused by prenatal THC exposure.

## Results

### Prenatal THC disrupts hippocampal-dependent memory

**Figure 1:**
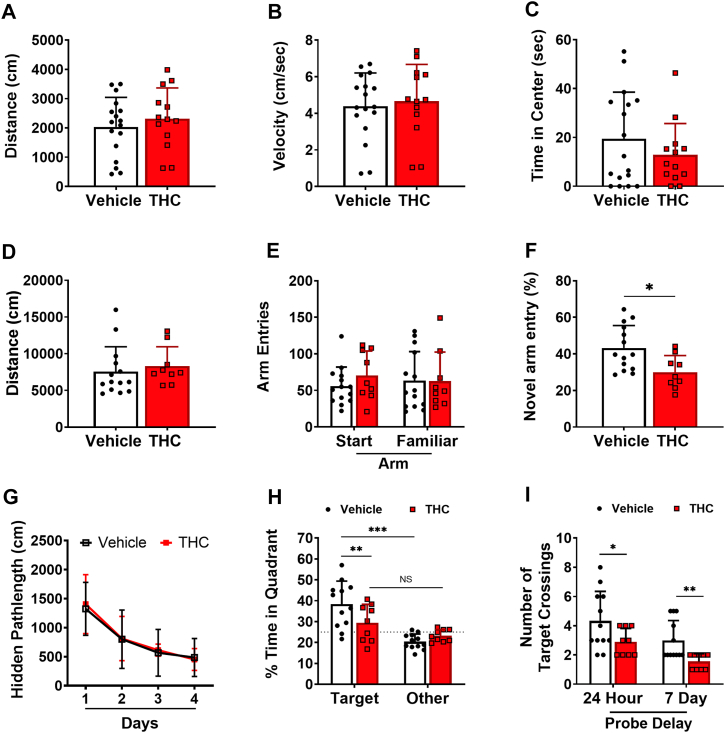
**Prenatal THC disrupts hippocampal-dependent memory.***A*, total distance traveled during the open field test. *B*, velocity during open field testing. *C*, time spent in the center area of the open field test. For *A*–*C*, n = 13 to 17/group. *D*, total distance traveled during training in the novel arm y-maze task. *E*, number of arm entries in the start and familiar arms during training in the novel arm y-maze task. *F*, percent entries into the novel arm during testing in the novel arm y-maze task. For *D*–*F*, n = 9 to 14/group. *G*, path length during hidden platform training in the Morris water maze. *H*, the percentage time in the target quadrant *versus* the average of the other three quadrants during a probe trial was performed 24 h after hidden path length training in the Morris water maze. *I*, the number of platform crossings during a probe trial performed 24 h (*left*) and 7 days (*right*) after hidden path length training in the Morris water maze. For *G*–*I*, n = 9 to 12/group. Mean ± SD; ∗*p* < 0.05, ∗∗*p* < 0.01, ∗∗∗*p* < 0.001. THC, delta-9-tetrahydrocannabinol.

We first sought to determine if there were any effects of prenatal THC exposure on general locomotor activity using the open-field task. There were no differences in the total distance traveled (t = 0.747, df = 28, *p* = 0.461; Fig. 1*A*) or mean speed/velocity (t = 0.388, df = 28, *p* = 0.701; Fig. 1*B*), suggesting prenatal THC exposure did not produce overt differences in locomotor activity. We also examined entries into the center portion of the arena during the open field task to assess anxiety-related behavior (33), as rodents tend to spend more time near the wall (34). Although rats exposed to THC tended to spend less time in the center, this effect was not significant (t = 1.07, df = 28, *p* = 0.148; Fig. 1*C*).

We next examined performance in the novel arm y-maze task, which examines spatial short-term memory. There were no differences in the total distance traveled (t = 0.575, df = 21, *p* = 0.572; Fig. 1*D*) or the number of arm entries for the start arm or familiar arm (t = 1.195, df = 21, *p* = 0.245 and t = 0.036, df = 21, *p* = 0.967, respectively; Fig. 1*E*) during the training session in which only two of the three arms could be explored. However, during the test trial in which the novel arm was open and could be explored, THC exposure reduced the percentage of entries into the novel arm relative to controls (t = 2.77, df = 21, *p* = 0.011; Fig. 1*F*). This reduction in novel arm exploration suggests that THC impairs the response to novelty and spatial memory in the y-maze.

We then used the Morris water maze (MWM) task to assess alterations in spatial learning and reference memory. During hidden platform training, the pathlength to reach the hidden platform during MWM acquisition did not differ between the groups (RMANOVA: Day∗Treatment [F(3,63) = 0.11, *p* = 0.956]; Fig. 1*G*). However, during the probe trial, which took place 24 h following the last training trial, impaired performance for THC-exposed animals was evident in a decreased time spent in the target quadrant relative to controls (t = 2.682, df = 38, *p* = 0.011; Fig. 1*H*). Whereas vehicle-exposed animals preferred the target quadrant relative to the average of the other three quadrants (t = 5.345, df = 13.35, *p* < 0.001; Welch’s *t* test), THC-exposed animals did not prefer the target quadrant (t = 1.968, df = 9.685, *p* = 0.078; Welch’s *t* test; Fig. 1*H*). Instead, THC animals exhibited chance performance, defined as 25% for percent time in the target quadrant. This deficit was also evident in the number of platform crossings, which was significantly decreased during the probe trial for THC-exposed animals 24 h after the last training session (t = 2.192, df = 16, *p* = 0.043; Welch’s *t* test; Fig. 1*I*, left). This deficit was still evident for platform crossings in the probe trial that occurred 1 week following the last training trial (t = 3.382, df = 15.08, *p* = 0.004; Welch’s *t* test; Fig. 1*I*, right).

### Prenatal THC impaired hippocampal basal glutamatergic synaptic transmission and synaptic plasticity

**Figure 2:**
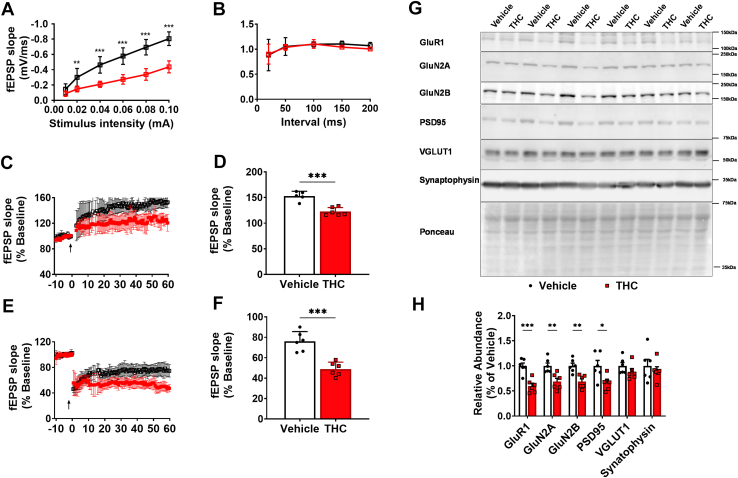
**Prenatal THC impaired hippocampal basal glutamatergic synaptic transmission and synaptic plasticity.***A*, input-output curve of fEPSP slope measured at increasing stimulus intensities. *B*, paired-pulse facilitation expressed as the ratio of the second stimulus fEPSP slope to the first stimulus fEPSP slope plotted as a function of interstimulus interval. *C*, long-term potentiation (LTP) graph represents fEPSP slope before and after induction by TBS normalized to baselines levels. *D*, LTP bar graph shows the average of fEPSPs recorded during the time period 50 to 60 min following TBS induction (*arrow*) normalized to baselines levels. n = 5 to 6 per group. *E*),long-term depression (LTD) graph represents fEPSP slope before and after induction by low-frequency stimulation (LFS) (*arrow*) normalized to baseline levels. *F*, LTD bar graph shows fEPSPs recorded for 50 to 60 min following LFS induction normalized to baseline levels. n = 6 per group. Mean ± SD. *G*, representative immunoblots showing protein levels of GluN2A, GluN2B, GluR1, PSD95, VGLUT1, and synaptophysin in hippocampal lysates from vehicle- and THC-exposed offspring. Ponceau S staining is shown as the total protein loading control. *H*, quantification of glutamatergic synaptic proteins expressed as relative abundance normalized to vehicle controls' total protein. GluN2B, GluN2A, and GluR1 protein levels are significantly reduced in the THC group, whereas VGLUT1 and synaptophysin levels are not significantly altered. Data are presented as mean ± SD. Individual data points represent biological replicates. ∗*p* < 0.05, ∗∗*p* < 0.01, ∗∗∗*p* < 0.001 *versus* vehicle. n = 6 animals per group. fEPSP, field excitatory postsynaptic potential; THC, delta-9-tetrahydrocannabinol.

To determine the physiological basis of the observed behavior changes, we next examined changes in basal synaptic transmission by comparing the slope of fEPSP across a range of stimuli and observed that prenatal THC exposure significantly decreased fEPSP slopes as stimulus intensities increased (RMANOVA: Intensity∗Treatment [F(5,50) = 27; *p* < 0.001]; Fig. 2*A*). To determine whether alterations in basal synaptic transmission could be related to alterations in presynaptic glutamate availability, we evaluated paired-pulse facilitation, a type of short-term plasticity that depends on residual calcium build-up in the presynaptic terminal. We observed no significant change in paired-pulse facilitation (RMANOVA: Interval∗Treatment [F(4,52) = 0.39; *p* = 0.813]; Fig. 2*B*), suggesting presynaptic release probability did not differ.

To determine whether THC alters synaptic plasticity, we measured LTP and LTD in the Schaeffer collateral pathway using hippocampal slices. The fEPSP slope, as a percentage of baseline, showed a significant decrease by ∼20% in LTP maintenance of THC-exposed animals compared to controls (t = 5.67, df = 10, *p* < 0.001; Fig. 2*D*). In a separate set of experiments, LTD was compared between the groups, and a reduction in the fEPSP slope by >25% from baseline in control slices as compared to a >50% reduction in THC slices was observed (t = 5.67, df = 10, *p* < 0.001; Fig. 2*F*). Together, these results demonstrate that prenatal THC exposure results in a reduction in LTP and enhancement of LTD in adolescence, indicating a persistent bias toward synaptic weakening within hippocampal circuits critical for memory formation.

Next, we investigated whether the changes in hippocampal basal glutamatergic neurotransmission were associated with changes in glutamate receptors, postsynaptic scaffold proteins, or presynaptic vesicle markers (Fig. 2, *G* and *H*). THC animals exhibited decreases in GluR1 (t = 5.00, df = 10, *p* < 0.001), GluN2A (t = 3.57, df = 10, *p* = 0.005), GluN2B (t = 4.16, df = 10, *p* = 0.002), and PSD95 (t = 2.72, df = 10, *p* = 0.021). In contrast, prenatal THC exposure did not affect the presynaptic vesicle markers VGLUT1 and synaptophysin. Together, these results indicate that prenatal THC exposure reduces postsynaptic glutamate receptor and scaffold protein expression without affecting presynaptic markers, consistent with a postsynaptic mechanism underlying the observed deficits in hippocampal synaptic plasticity.

### THC exposure impairs AMPAR-mediated synaptic transmission but increases NMDAR-mediated synaptic transmission with an overall shift toward inhibitory currents

**Figure 3:**
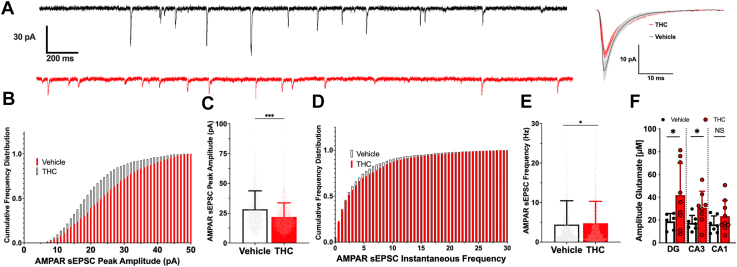
**Prenatal THC exposure impairs AMPAR-mediated synaptic transmission.***A*, representative traces of AMPAR current from vehicle- and THC-exposed rodents with average trace for all events and their SEM plotted as colored shadow (*black*–vehicle, *red*–THC). *B*, cumulative frequency distribution curve for AMPAR sEPSC peak current amplitude. *C*, AMPAR sEPSC peak amplitude. *D*, cumulative frequency distribution curve for AMPAR sEPSC instantaneous frequency. *E*, frequency of sEPSCs for AMPAR currents. For *B*–*E*, n = 4 to 6 cells. *F*, KCl-evoked glutamate release in the DG, CA3, and CA1 hippocampal subregions. n = 6 to 9 animals. Mean ± SD; ∗*p* < 0.05, ∗∗*p* < 0.01, ∗∗∗*p* < 0.001. CA1, cornu ammonis 1; CA3, cornu ammonis 3; DG, dentate gyrus; sEPSC, spontaneous excitatory postsynaptic current; THC, delta-9-tetrahydrocannabinol.

Since long-term plasticity was biased toward synaptic weakening in THC-exposed rodents, we next compared spontaneous excitatory postsynaptic currents (sEPSCs) recorded from cornu ammonis 1 (CA1) pyramidal cells in acute slices from vehicle and THC groups to investigate the cellular mechanism underlying the altered plasticity. Electrically isolated AMPAR currents were measured from each group. We observed a decrease in AMPAR-mediated sEPSC peak amplitude (KS = 0.24, *p* < 0.001; Fig. 3, *A*–*C*) and an increase in AMPAR sEPSC frequency (KS = 0.07, *p* = 0.033; Fig. 3, *D* and *E*) for THC-exposed animals. This observed decrease in amplitude but increase in frequency suggests that the number of AMPARs was decreased, but presynaptic stimulation for synaptic communication was increased (35, 36, 37). This could be due to THC mediated early effect that could be the basis of shift in E/I balance caused by hypersynchronous release frequency and AMPAR activation, leading to eventual desensitization and internalization of these receptors. Our data from MEA studies recording release properties and how they are linked with postsynaptic responses show a decreased vesicular release with increased quanta of release. Specifically, KCl-evoked release of glutamate was increased in the dentate gyrus (DG) (t = 2.367, df = 9.424, *p* = 0.041; Welch’s *t* test) and cornu ammonis 3 (CA3) (t = 2.356, df = 10.09, *p* = 0.04; Welch’s *t* test) but not CA1 (t = 1.345, df = 12.70, *p* = 0.202; Welch’s *t* test) for THC-exposed animals (Fig. 3*F*). This presynaptic increase in the quantal release of glutamate is in line with the sEPSC data, as it might have caused desensitization of the AMPAR population and resulted in a shift toward altered E/I balance.

**Figure 4:**
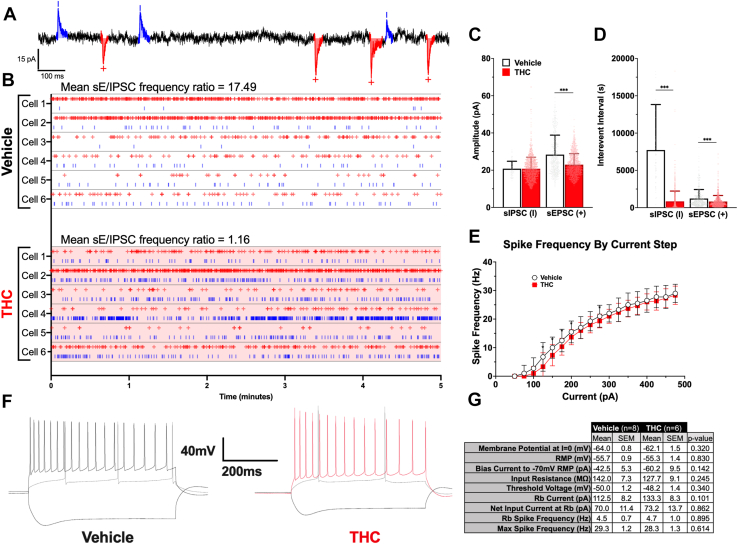
**Alteration in E/I balance, as recorded from complex synaptic events in THC-exposed rodents, shows a shift toward inhibitory drive.***A*, representative complex traces of AMPAR + NMDAR/GABA (E/I) currents from vehicle-exposed rodents. Upward (*blue*) deflections represent GABA-mediated sIPSCs. Downward (*red*) deflections represent AMPA + NMDAR-mediated sEPSCs. *B*, Raster plot of sEPSCs (+) and sIPSCs (I) during the same 5-min recording period for six cells from each treatment group (vehicle and THC). *C*, mean sIPSC and sEPSC amplitudes. *D*, the mean interevent interval for the vehicle and THC groups. *E*, spike frequency plot with increased steps of current compared between the vehicle and THC-exposed groups. *F*, raw traces from such current-step recordings. *G*, table depicting intrinsic properties of all recordings. Mean ± SD; ∗∗∗*p* < 0.001. n = 6 for each comparison. RMP, resting membrane potential, Rb, rheobase current; note that Rb and Max spike frequency remained constant. E/I, excitation/inhibition; GABA, gamma-aminobutyric acid; sEPSC, spontaneous excitatory postsynaptic current; sIPSC, spontaneous inhibitory postsynaptic current; THC, delta-9-tetrahydrocannabinol.

Shifts in AMPAR receptor signaling through desensitization can be linked to network hyperexcitability and result in altered excitation/inhibition (E/I) balance (24, 38). Therefore, to study overall network excitability, we also performed current-clamp and patch-clamp recordings to measure the intrinsic membrane and firing properties of CA1 pyramidal neurons as well as record complex traces with both excitation and inhibition components (Fig. 4). To study the relative contribution of E/I balance (mediated by GABA_A_Rs and NMDAR/AMPARs), we examined the complex traces. Fig. 4*A* shows a sample trace from a vehicle-exposed animal in which upward deflections from baseline reflect GABA-mediated spontaneous inhibitory postsynaptic currents (sIPSCs) while downward deflections represent AMPAR/NMDAR-mediated sEPSCs. For these complex spontaneous recordings, cells were held at −40 mV to record unstimulated postsynaptic responses. The time of peak values for postsynaptic currents was used to generate an E/I raster plot representing the frequency of events over 5 min of recording (Fig. 4*B*) as previously described (39). Our data suggest an increase in sIPSCs in THC-exposed rodents compared to the vehicle group (vehicle mean sE/IPSC ratio: 17.49 *versus* THC mean sE/IPSC ratio 1.16; Fig. 4*B*) as seen in the Raster plot analysis of sEPSCs (+) and sIPSCs (I) during the same period. The THC group showed no change in mean sIPSC (KS = 0.1707, *p* = 0.079; Fig. 4*C*, left) but significantly lower mean sEPSC amplitude (KS = 0.2418, *p* < 0.001; Fig. 4*C*, right). The THC-exposed group also exhibited a significantly higher mean sIPSC frequency compared to the vehicle group (KS = 0.8414, *p* < 0.001; Fig. 4*D*, left), as well as a decrease in sEPSC frequency (KS = 0.1833, *p* = <0.001; Fig. 4*D*, right), suggesting an overall increase in inhibitory drive in these synapses. Spike frequency was significantly lower in the THC group at certain points (Mixed Model: Treatment [F(1,216) = 9.275, *p* = 0.003], Fig. 4*E*). This signifies that at this age point, THC exposure caused changes in excitability and threshold of excitation for these synapses, which in turn will alter the E/I balance at a cellular level. The embedded table (Fig. 4*G*) shows the characteristics and intrinsic properties of cells recorded in the current-clamp configuration and do not reveal significant differences in their firing properties.

**Figure 5:**
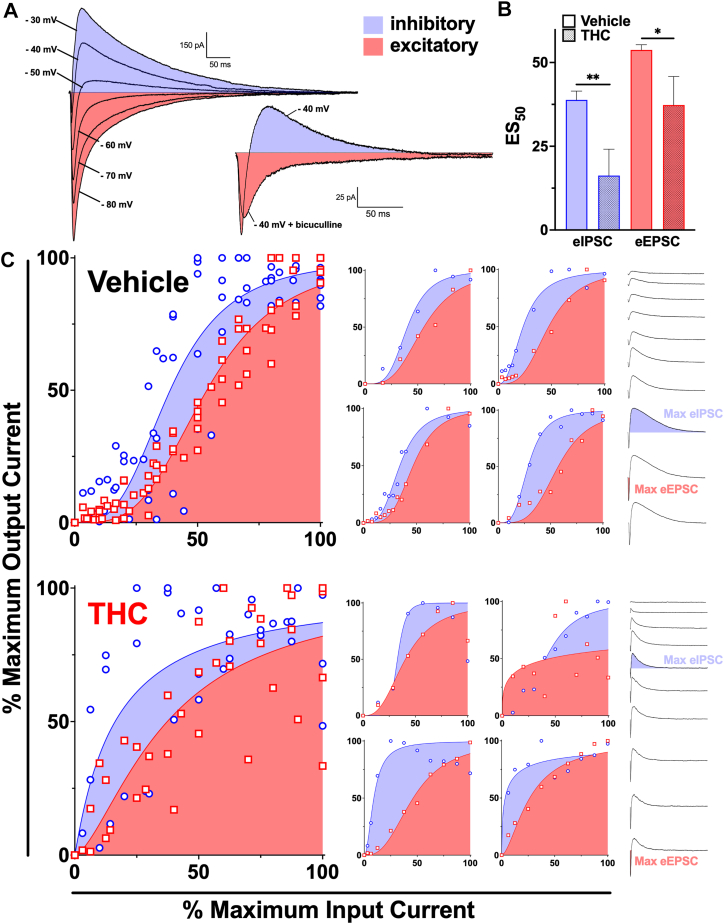
**PCE alters presynaptic recruitment of inhibitory and excitatory inputs.***A*, representative traces of evoked compound E/IPSCs demonstrating the voltage-current relationship of each component at a fixed stimulus intensity. *Blue* and *red* represent the inhibitory and excitatory components of the compound trace respectively. Bicuculline blocks the inhibitory GABAergic component of the compound postsynaptic response. *B*, comparison of mean ES50 values for each eIPSC and eEPSC between pooled vehicle (*solid* fill) and THC (*dotted* fill) cells (n = 7 vehicle, 4 THC; Mean ± SD; ∗∗*p* < 0.01, ∗*p* < 0.05). *C*, normalized stimulation-response curves of evoked eE/IPSCs fitted with nonlinear regression. Large graphs contain pooled data from all recorded cells in each group (n = 7 vehicle, 4 THC). Smaller inset graphs contain data from individual cells within each group. eE/IPSC traces to right of graphs depict degrees of separation between stimuli needed to elicit max eIPSC and max eEPSC components of the compound trace. The trace in which each component reached its maximum is labeled and filled to the *x*-axis with the corresponding inhibitory/excitatory color. GABA, gamma-aminobutyric acid; IPSC, inhibitory postsynaptic current; THC, delta-9-tetrahydrocannabinol.

To further understand how changes in neuronal connectivity may be contributing to observed differences in spontaneous activity, electrically evoked compound excitatory-inhibitory postsynaptic currents (eE/IPSCs) were isolated and analyzed (Fig. 5*A*). These data were graphed as percent maximum input current (stimulation) and percent maximum output current (eE/IPSC amplitude). Normalized input-output (I/O) values comparing stimulation intensity to eE/IPSC amplitude mimicked distributions of standard dose-response curves, demonstrating a central linear range bordered by early “lag” and late “plateau” phases (Fig. 5*C*). Attempting to conserve terminology conventions from dose-response analyses, effective “stimulation” (*in lieu* of “concentration”) values that elicited 50% of maximum response (ES_50_) were compared between groups for each component of the compound postsynaptic response (Fig. 5*B*).

In tandem with increases in the frequency of both sEPSC and sIPSC events in the THC group, lower evoked E/IPSC ES_50_ values possibly indicate higher baseline engagement of the presynaptic network, with potentially more active and/or readily excitable individual synapses. Individual cells within each group (Fig. 5*C* inset graphs) maintained sigmoidal distributions for E/I I/O relationships, though cells in the THC group exhibited a strong leftward shift in the evoked IPSC (eIPSC) component. Collectively, E/I I/O relationships are visibly altered by prenatal cannabinoid exposure. Pooled data from vehicle cells maintains sigmoidal distribution, while data pooled from THC cells demonstrates a left and upward shift in both excitatory and inhibitory components of the stimulation-response curves. This bowing shift seems to abolish the central linear range, and the stimulation needed to elicit 50% of the maximal inhibitory response is less than half that of control animals (eIPSC EC50 38.83 *versus* 16.26; t = 3.360, df = 9, *p* = 0.0084). eEPSC ES50 values were also significantly different between Vehicle and THC groups (eEPSC EC50 53.75 *versus* 37.30; t = 2.522, df = 9, *p* = 0.0326). To further aid in the visualization of this phenomenon, sample traces from each group depict the separation in maximal eIPSC and eEPSC recruitment (Fig. 5*C*). Cells from THC animals reached maximum eIPSC amplitude with far less stimulation than those from vehicle-treated animals, and eEPSC amplitude continued to increase following a plateau of the inhibitory component of the postsynaptic response. Together, these results suggest a significant decoupling of inhibitory and excitatory neurotransmission following THC exposure in utero. This decoupling is predictably occurring presynaptically, as eE/IPSC experiments largely rely on the recruitment of direct inputs to the recorded cell and stimulation of its localized network.

### THC exposure alters CB_1_ and VGAT expression across hippocampal subregions

To understand the molecular basis of the observed physiological changes in sIPSCs, we next studied the spatial expression of specific inhibitory proteins known to support inhibitory synaptic function to determine how sIPSCs occur. Previous studies have established that (i) increases in sIPSC frequency typically reflect enhanced presynaptic GABA release (40) and (ii) that CB_1_Rs are enriched on VGAT-positive interneuron terminals to regulate GABA release (41). We therefore quantified CB_1_R and VGAT expression to determine whether prenatal THC exposure alters inhibitory presynaptic molecular mechanisms contributing to shifts in E/I balance. We measured CB_1_R expression relative to VGAT by immunohistochemistry (IHC) in vehicle- and THC-exposed offspring. To establish where THC exposure may alter the hippocampal circuitry, we further separated IHC hippocampal images into three regions: (i) DG, (ii) CA3, and (iii) CA1.

**Figure 6:**
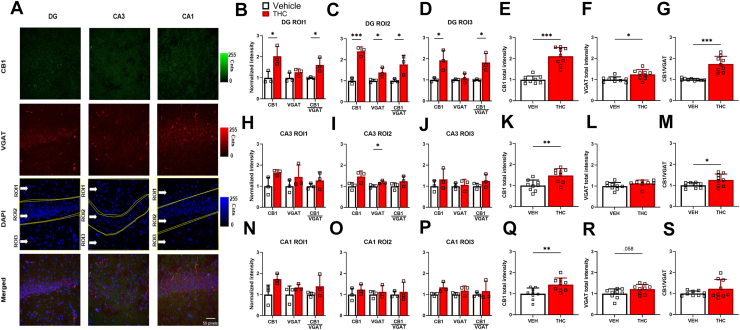
**PCE alters CB_1_ and VGAT expression across hippocampal subregions.***A*, representative images of CB_1_ (*green*), VGAT (*red*), DAPI (*blue*), and merged (*bottom*). *Dotted outlines* indicate three regions of interest (ROI1–ROI3) within each hippocampal subregion. (the scale bar represents 5 pixels). *B*–*G*, CB_1_R total expression, VGAT total expression, and CB_1_R/VGAT ratios in the DG were quantified across individual ROIs and aggregated across ROIs. *H*–*M*, CB_1_R, VGAT, and CB_1_R/VGAT ratios in CA3 were quantified across ROIs and aggregated values. *N*–*S*, CB_1_R, VGAT, and CB_1_R/VGAT ratios in CA1 were quantified across ROIs and aggregated values. All values are normalized to vehicle. n = 3/group. Mean ± SD; *p* < 0.05, ∗*p* < 0.01, ∗∗*p* < 0.001; statistical comparisons performed using unpaired or Welch’s *t*-tests as appropriate. CA3, cornu ammonis 3; CB_1_R, cannabinoid receptor type 1; DAPI, 4′,6-diamidino-2-phenylindole; DG, dentate gyrus; ROI, region of interest; VGAT, vesicular GABA transporter.

To isolate the specific portion of the circuit connections affected by THC exposure, we separated each region (DG, CA3, and CA1) into three sub-regions of interest (ROIs) (Fig. 6, *B*–*D*, *H*–*J*, and *N*–*P*). Within the DG (Fig. 6, *B*–*D*), ROI1 was selected based on the area receiving Schaffer collateral projections, ROI2 encompassed regions influenced by granule cell input, and ROI3 aligned with the zone where mossy fibers project toward CA3. For CA3 (, *H*–*J*), ROI1 was placed within the area receiving medial septal input (42), ROI2 corresponded to the Schaffer collateral pathway (43, 44), and ROI3 captured the arrival of mossy fiber projections from DG granule cells (43). In CA1 (CA1; Fig. 6, *N*–*P*), ROI1 was selected to represent the cortex outside the hippocampus (45), ROI2 was the Schaffer collateral pathway (43), and ROI3 included regions receiving convergent temporoammonic and perforant path inputs from the entorhinal cortex (43).

Although THC exposure altered CB_1_R and VGAT expression across all hippocampal subregions the greatest increases occured in the DG region resulting in significantly increased CB_1_R expression across all ROIs, with increases of ∼100% in ROI1 (t = 3.2, df = 4, *p* = 0.033; Fig. 6*B*), R02 (t = 9.5, df = 4, *p* = 0.0001; Fig. 6*C*), and R03 (t = 3.3, df = 4, *p* = 0.030; Fig. 6*D*). Meanwhile, VGAT expression was ∼40% increased in ROI2 (t = 2.8, df = 4, *p* = 0.046; Fig. 6*C*), but not significantly different in ROI1 or ROI3. The increased CB_1_R and VGAT expression led to a significant increase in the CB_1_/VGAT ratio across all ROIs (t = 3.2, df = 4, *p* = 0.034; Fig. 6*B*), (t = 3, df = 4, *p* = 0.041; Fig. 6*C*), and (t = 3.3, df = 4, *p* = 0.029; Fig. 6*D*). Aggregate DG CB_1_R expression increased by ∼100% (t = 7.5, df = 10.8, *p* < 0.001; Welch’s *t* test; Fig. 6*E*), VGAT expression increased by ∼25% (t = 2.9, df = 16, *p* = 0.012; Fig. 6*F*), resulting in an increase of ∼75% in the CB_1_R/VGAT ratio (t = 6, df = 8.3, *p* = 0.001; Welch’s *t* test; Fig. 6*B*). Together, these DG THC-exposure induced increases in CB_1_R expression and the inhibitory terminal marker VGAT suggest that inhibitory signaling within the DG is altered supporting the observed shift in E/I balance from electrophysiological results (Figure 3, Figure 4, Figure 5).

In the CA3, THC exposure did not alter CB_1_R expression across individual ROIs but significantly increased VGAT expression in ROI2 by ∼20% (t = 3, df = 4, *p* = 0.041; Fig. 6*I*). Aggregated CA3 data show a significant increase in CB_1_R expression by ∼50% (t = 3.5, df = 16, *p* = 0.003; Fig. 6*K*), leading to a higher CB_1_R/VGAT ratio compared to the vehicles (t = 2.5, df = 11, *p* = 0.030; Welch’s *t* test; Fig. 6*M*) across the CA3. Although the only significant change in CA3 was a modest increase in VGAT in ROI2, this localized shift may interact with the strong DG-to-CA3 projection, suggesting that even subtle CA3 changes could contribute to an altered E/I balance initiated in the DG.

In the CA1, THC exposure did not significantly affect CB_1_R or VGAT expression across individual ROIs (N-P). However, aggregated CA1 data show a significant increase in CB_1_R intensity (t = 1.3, df = 8, *p* = 0.006; Fig. 6*Q*) and a trend of increasing VGAT intensity was observed (t = 1, df = 8, *p* = 0.058; Fig. 6*R*). Although the CA1 showed only a modest increase in CB_1_R and a trend toward elevated VGAT, these findings suggest subtle alterations in inhibitory terminal composition within CA1.

**Figure 7:**
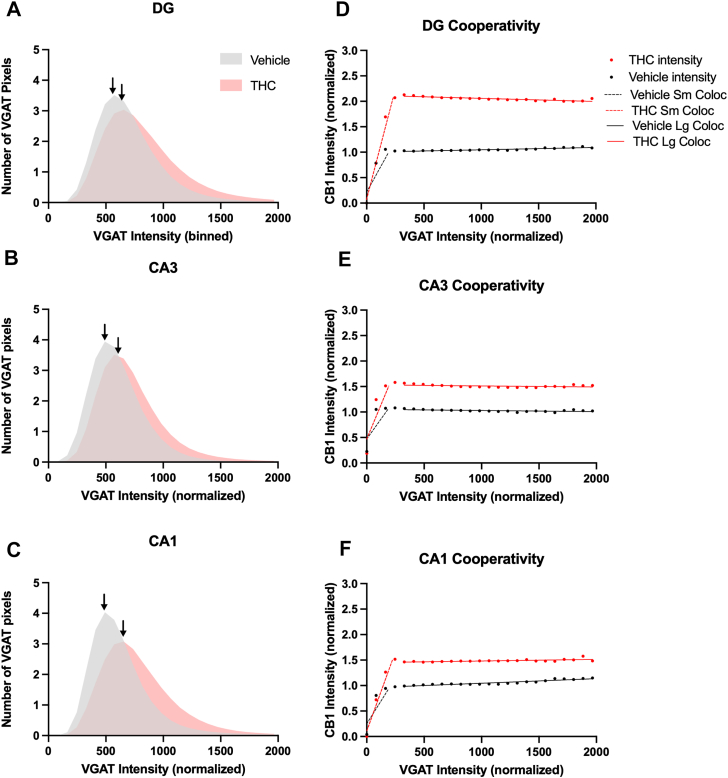
**THC alters vGAT intensity distributions and CB_1_ cooperativity across hippocampal subregions.***A*–*C*, the number of VGAT-positive pixels plotted across normalized vGAT intensity bins in the DG (*A*), CA3 (*B*), and CA1 (*C*) for vehicle (*gray*) and THC (*red*) groups. Shaded areas represent total VGAT pixels per VGAT bin, and *arrows* denote peak total VGAT intensity. *D*–*F*, CB_1_R cooperativity curves plotted as normalized CB_1_R intensity as a function of normalized VGAT intensity in DG (*D*), CA3 (*E*), and CA1 (*F*). *Dashed lines* represent small synapse colocalization regions (Sm Coloc), whereas solid lines indicate large synapse regions (Lg) with a fixed amount of CB1. Vehicle is shown in *black* and THC in *red*. Data are normalized to vehicle. n = 3 per group. CA3, cornu ammonis 3; CB_1_R, cannabinoid receptor type 1; DG, dentate gyrus; THC, delta-9-tetrahydrocannabinol; VGAT, vesicular GABA transporter.

Since CB_1_R regulates GABA release at the synaptic level, both total expression and distribution of CB_1_R relative to VGAT can affect the E/I balance observed electrophysiologically. However, mean intensity and ratio measurements alone cannot capture the organization of CB1R across inhibitory terminals. To understand how the change in total CB_1_R expression is distributed across synapses, we next examined intensity-level distributions of CB_1_R–VGAT to assess colocalization across the full VGAT expression range. This measurement will assess whether CB1R expression increases along with VGAT expression on a per-synapse basis. In the DG (Fig. 7*A*), THC exposure altered the distribution of VGAT intensities, with peak VGAT intensity shifted to the right relative to the vehicles, indicating that VGAT became concentrated within a smaller subset of inhibitory synapses rather than being uniformly distributed across many terminals. In the CA3 (Fig. 7*B*), THC exposure produced a similar but less pronounced shift in the distribution. In CA1 (Fig. 7*C*), THC exposure induced the largest redistribution, with a pronounced rightward shift in peak VGAT intensity, consistent with synaptic pruning of inhibitory synapses accompanied by increased VGAT content within the remaining synapses. These results show that the increase in VGAT expression is caused by a distribution of larger inhibitory synapses rather than simply more synapses overall.

We next examined CB_1_R colocalization with VGAT by plotting normalized CB_1_R intensity as a function of normalized VGAT intensity to determine whether THC exposure altered the association of CB_1_R with VGAT-positive inhibitory terminals (Fig. 7, *D*–*F*). We observed that as VGAT intensity increased, CB_1_R intensity initially increased at the same rate up to a fixed VGAT level (∼300 cnts/pixel for vehicle (VEH) and ∼400 cnts/pixel for THC), above which CB_1_R no longer scaled proportionally with VGAT. Combined with the increase in VGAT distribution, this plateau of CB_1_R intensity indicates that as VGAT-expressing synapses increase in size, the CB_1_R colocalization saturates for inhibitory terminals in the THC-exposed animals. This effect was evident in the initial slope of the DG (VEH: slope = 0.041 ± 0.00159 SE *versus* THC: slope = 0.009 ± 0.00097 SE; Fig. 7*A*). It was less pronounced in CA3 (VEH: slope = 0.003 ± 0.00171 SE *versus* THC: slope = 0.005 ± 0.00197 SE; Fig. 7*B*). The most observed difference in CB_1_R intensity increase as a function of VGAT intensity was most prominent in CA1 (VEH: slope = 0.004 ± 0.00149 SE *versus* THC: slope = 0.006 ± 0.00091 SE Fig. 7*C*) in the small synapse colocalization regions.

Further separation of large synapse regions showed that THC reduced CB_1_R recruitment to VGAT-positive terminals while elevating baseline CB_1_R signal across inhibitory terminals, consistent with diffuse CB_1_R expression rather than selective enrichment at large synapses. This was evident in the DG (VEH: y-intercept = 1.001 ± 0.00580 SE *versus* THC: y-intercept = 2.120 ± 0.0104 SE; Fig. 7*D*), CA3 (VEH: y-intercept = 1.053 ± 0.00916 SE *versus* THC: y-intercept = 1.533 ± 0.01285 SE; Fig. 7*E*) and CA1 (VEH: y-intercept = 0.956 ± 0.01018 SE *versus* THC: y-intercept = 1.450 ± 0.01045 SE; Fig. 7*F*) Together, these findings suggest that although total CB_1_R expression increases due to THC exposure, its localization to VGAT-positive inhibitory synapses is diminished, which supports the hypothesized reduced endocannabinoid control over VGAT-positive terminals previously observed and thereby promote the higher sIPSC frequency we observe in electrophysiological results (Figure 3, Figure 4, Figure 5).

Together, these findings demonstrate that prenatal THC exposure alters both the magnitude and organization of inhibitory signaling across hippocampal circuits. The DG exhibited the largest overall increases in CB_1_R and VGAT expression, whereas CA1 displayed the strongest redistribution of CB_1_R across inhibitory terminals. Although total CB_1_R levels were elevated, distributional analyses revealed that CB_1_R expression became more diffuse and less selectively concentrated at VGAT-positive synapses, indicating reduced endocannabinoid regulation of inhibitory terminals. CA3 and CA1 showed more modest changes in overall expression, but CA1 in particular exhibited a pronounced shift toward altered CB_1_R organization, suggesting region-specific vulnerability in inhibitory circuit regulation. Collectively, these coordinated changes in inhibitory terminal composition and CB_1_R localization provide a mechanistic framework for the elevated sIPSC frequency observed and point to disrupted E/I balance as a central consequence of prenatal THC exposure.

## Discussion

To our knowledge, this study is the first to integrate behavioral, electrophysiological, molecular, and subregional localization analyses to demonstrate that prenatal THC exposure produces a hippocampal E/I imbalance during adolescence. Furthermore, this current study demonstrates that prenatal THC exposure causes deficits in synaptic plasticity and hippocampal-based memory that can persist into the adolescent period of rodent offspring. Both LTP and LTD, measures of plasticity, are impaired in THC-exposed rodents, indicating a significant shift in plasticity in this exposure regimen. Consistent with these network deficits, key NMDA and AMPA receptor subtypes involved in LTP and LTD induction and maintenance were also decreased. These molecular alterations provide a structural basis for the observed cellular impairments in excitatory transmission. THC rodents exhibited decreased AMPAR-mediated sEPSC amplitude, suggesting a relative decrease in AMPAR current and a shift toward non-AMPAR signaling contribution in synaptic communication in the hippocampal Schaffer collateral pathway. These data are in line with the impaired plasticity observed in THC animals. Presynaptic release measurement studies suggest a decrease in vesicular release frequency with increased release quanta. We also observed a shift of activity toward non-AMPAR-mediated signaling from AMPAR-mediated signaling, a manifestation of long-term changes as a result of THC exposure. Altered activity may be linked to network hyperexcitability and disrupt E/I balance. Our data suggest an increase in sIPSCs and an overall increase in inhibitory drive in hippocampal synapses of THC-exposed animals. Spike frequency was also significantly lower in the THC group. Although total CB_1_R expression was increased following prenatal THC exposure, distribution analyses indicated reduced colocalization with VGAT-positive inhibitory terminals. This reorganization may weaken endocannabinoid regulation of GABA release and help explain the elevated sIPSC frequency observed in adolescent hippocampal neurons. Taken together, these data suggest that THC exposure causes changes in excitability and threshold of excitation for these synapses, ultimately altering the E/I balance.

For the current study, we used a dose of 5 mg/kg of THC, which corresponds to moderate cannabis exposure in humans after correction for differences in body surface area (46). This dose was previously shown not to cause overt abnormalities, including no alterations in maternal weight, fetal weight, litter size, gestation time, or pup mortality (47, 48). This dose, rather than a low dose, also aligns with the current trends of increasing potency and usage levels (3, 49, 50). We started treatment at gestational day 5 because earlier administration can lead to spontaneous abortion (51) and extended treatment to postnatal day (PND) 9 because the third trimester in humans corresponds to an early postnatal period in rats (reviewed in (52)). Based on peak NMDA receptor density and synaptogenesis, which are estimated to occur around PND 6 to 9 (53, 54), we have chosen a treatment paradigm in which the last day of treatment is PND 9, a regimen validated in other studies of prenatal THC exposure (47, 48). Because we are interested in not only the effects of THC that crosses the placenta, resulting in fetal exposure, but also that passed *via* breast milk, resulting in neonatal exposure (55), we opted not to cross-foster PCE pups to untreated dams at birth to allow for continued exposure until PND 9. However, in future studies, we will delineate the consequences of prenatal and postnatal factors by cross-fostering PCE pups.

Rats reach puberty at an average age of 50 days after birth (PND 50) (56). We opted to examine the consequences of PCE during the periadolescent period (PND 40–65) to facilitate comparisons with the human literature, in which adolescence is one of the most commonly studied periods following perinatal exposure. In addition, this time period predicts adulthood outcomes (57, 58), suggesting that deficits during the periadolescence period may have lasting consequences. In future studies, we will also examine the long-term consequences of prenatal cannabinoid exposure on adulthood, specifically aging, when the addition of age-related insults may reveal latent PCE-mediated deficits. Males were selected for the current study because prior studies indicated that males, but not females (11, 59), exhibit deficits in synaptic plasticity (59), hippocampal function (11, 25, 60), and spatial memory (11, 61) following prenatal cannabinoid exposure. However, a more recent study revealed spatial memory deficits in females (62) and suggests future studies should address sex as a biological variable.

Observational human studies have demonstrated that prenatal cannabinoid exposure results in cognitive impairments, including impairments in memory, analysis, and attention, during the adolescent period (17, 18). In the current study, we observed no differences in locomotion during the open-field test for THC-exposed animals. This finding is consistent with some prior studies (9, 22), though a lower THC dose (2 mg/kg) for a shorter duration (gestational days 5–20) was shown to increase total locomotion in the open field (61). In the current study, no differences were observed in time spent in the center of the open field, consistent with prior studies (61). We also observed deficits in hippocampal-dependent memory in THC-exposed animals in both the novel-arm y-maze and MWM tasks, similar to previous reports (9, 11, 61). Interestingly, we did not observe differences in MWM acquisition, as evidenced by similar performance during hidden platform testing, despite differences during probe trials assessing spatial reference memory. THC-exposed animals likely used a nonspatial search strategy, such as a chaining strategy, which is effective when the hidden platform is available but ineffective when it is removed (63, 64). Future studies should further evaluate the learning strategy used following prenatal THC exposure (65).

To determine whether behavioral impairments in THC-exposed animals might be linked to alterations in glutamatergic neurotransmission, we examined basal synaptic transmission and synaptic plasticity in acute hippocampal slices. We observed a decrease in basal synaptic transmission, likely due to reduced glutamate binding to synaptic AMPA receptors and increased GABAergic inhibition, as supported by decreased GluA1 levels and increased sIPSC frequency. These deficits also likely contributed to the synaptic plasticity deficits observed in PCE animals, given that concurrent activation of the glutamatergic receptors of both AMPA and NMDA subtypes is needed for LTP induction (67, 68), and appropriately tuned GABAergic inhibition is also required (66). In addition to its role in LTP induction, GABAergic signaling also regulates short-term plasticity processes such as post tetanic potentiation, a transient enhancement of presynaptic release that precedes LTP maintenance, and short-term depression, a temporary reduction in synaptic efficacy that contributes to LTD maintenance (67, 68). Although we did not directly assess post tetanic potentiation or short-term depression, altered inhibitory drive in THC-exposed animals may significantly influence these short-term plasticity dynamics and warrants investigation in future studies.

Decreased LTP induction in PCE offspring also suggests reduced activation of NMDARs, particularly synaptic GluN2A-containing NMDARs, which is supported by our findings of reduced hippocampal GluN2A in THC-exposed animals. In addition, we observed decreased levels of PSD95, a core scaffolding protein that anchors and clusters NMDA receptors at synaptic sites. This could further explain compromised synaptic GluN2A signaling (20) and exacerbate alterations in GluN2B-dependent plasticity pathways previously reported (61). Despite the postsynaptic alterations observed, unchanged VGLUT1 and synaptophysin indicate preserved presynaptic structure, whereas region-specific increases in KCl-evoked glutamate release suggest functional rather than structural presynaptic alterations. Overall, we saw a reduction in LTP and enhancement of LTD, suggesting an impairment of plastic events in the hippocampus following THC exposure. More studies are needed to further identify the role of isolated NMDAR current in this scenario. The observation of decreased sEPSC amplitude in these complex E/I trace experiments could be due to a mixed contribution of both AMPAR and NMDAR currents. In our data (Fig. 3), we observed a decreased AMPAR response when measured electrophysiologically as separate receptors (voltage-mediated recording). However, in recordings with both E/I components at −40 mV, the effects on AMPAR and NMDAR function would be mixed in the absence of any pharmacological blocker. Since AMPAR distribution and density are known to increase as synapses mature during development (69), the observed reduction in AMPA-mediated sEPSC amplitude also suggests that while individual synapses may be more active and/or more numerous, they are likely less mature. This is supported by eE/IPSC recordings, which show that numerous underdeveloped synapses can still summate under stimulation.

Taken together, these mixed excitatory effects point to a broader reorganization of synaptic balance rather than a simple reduction in excitatory signaling. Consistent with this interpretation, our data suggest an overall shift toward increased inhibitory drive, potentially reflecting consolidation of THC-mediated plasticity changes. Specifically, we observed altered AMPAR and NMDAR contributions, a decreased excitability threshold, and increased sIPSCs in the THC group. These findings motivated a more detailed interrogation of inhibitory circuit engagement within this paradigm. Comparison of ES_50_ values between vehicle and THC animals helped to further investigate how PCE may contribute to circuit disorganization during neuronal development. Our data suggest that prenatal THC exposure alters presynaptic recruitment dynamics, with inhibitory synapses becoming engaged at lower thresholds and losing coordinated scaling with excitatory inputs. Together, these alterations in recruitment thresholds and ES_50_ values indicate that prenatal THC exposure produces long-lasting disorganization of inhibitory and excitatory convergence onto CA1 pyramidal neurons, biasing synaptic integration toward inhibition during adolescence. Although prior studies reported that E/I balance is shifted toward an increase in excitation due to a reduction in GABAergic inhibition in the ventral tegmental area and cholecystokinin-expressing interneurons (70), our data indicate enhanced recruitment of inhibitory inputs and elevated sIPSC frequency in adolescent CA1 neurons, suggesting that prenatal THC can remodel inhibitory circuit function in a region-specific manner. In addition, our findings suggest that consistent with the prior study, VGAT-positive synapses may be reduced, but the remaining inhibitory synapses exhibit increased VGAT expression per synapse. The remodeling of the inhibitory circuit may be due to prior disruption in interneuron migration (71), projection neuron maturation (11), neurite outgrowth (72), neurogenesis (73), and reduced CB_1_R-VGAT colocalization (74) observed at earlier time points following prenatal cannabinoid exposure.

Such developmental perturbations would be expected to alter not only the expression and functionality of inhibitory synapses, but also the molecular organization of presynaptic regulatory machinery at these terminals. Consistent with this possibility, we observed increased CB_1_R expression during adolescence, consistent with prior mRNA data in males (75). Though our distribution analyses revealed reduced colocalization with VGAT-positive terminals, suggesting diminished endocannabinoid regulation of inhibitory synapses. In addition, a prior study reported increased CB_1_R functionality in the prefrontal cortex (84), but even though we did not directly assess CB_1_R functionality in the hippocampus, the discrepancy could be due to differences in brain region, and the possible increased CB_1_R functionality may not be acting on VGAT-positive terminals, as our data show. Reduced localization of CB_1_Rs at VGAT-positive terminals in the hippocampus would be expected to relieve endocannabinoid-mediated suppression of GABA release (76), thereby increasing presynaptic release probability and contributing to the elevated sIPSC frequency observed electrophysiologically. Interestingly, sIPSC amplitude remained unchanged, but evoked presynaptic recruitment showed the most profound alteration. Disorganization and reduced CB_1_R-VGAT colocalization might also suggest that localized stimulation does not recruit concentrated circuits of inhibitory inputs, as it would in a tightly organized system, but instead maximally stimulates diffuse presynaptic inhibitory inputs to the recorded cell.

These observed deficits in learning and memory are supported by our IHC findings. Our data indicate that the DG was the hippocampal subregion most vulnerable to THC-induced changes in CB_1_R expression. This heightened sensitivity may reflect the high density of cholecystokinin-expressing interneurons in the DG, which are enriched for both VGAT and CB_1_Rs and preferentially mediate dendritic inhibition (41). In contrast, parvalbumin-expressing interneurons, which are particularly abundant in CA1, typically lack CB_1_Rs and provide fast, temporally precise perisomatic inhibition that tightly regulates the initiation of action potentials (77, 78), which may explain why the CA1 region exhibited the largest diffuse CB_1_R recruitment to VGAT-positive terminals. Because inhibitory architecture differs across hippocampal subregions, with predominantly dendritic inhibition in the DG and a mixture of dendritic and perisomatic inhibition in CA1 and CA3, region-specific CB_1_R disruption is likely to have distinct functional consequences. Preferential loss of CB_1_Rs at dendrite-targeting boutons would be expected to weaken feedforward dendritic inhibition in the DG and in dendritic layers of CA1 and CA3 more than perisomatic inhibition. This loss could alter spike timing and firing probability, thereby destabilizing E/I balance. Therefore, given that parvalbumin and cholecystokinin interneurons can affect sIPSC frequency and amplitude (79, 80), future studies should investigate the density of parvalbumin and cholecystokinin interneurons in this paradigm.

Mechanisms of LTP/LTD are heavily reliant on strictly controlled inhibitory inputs (81), and interference with CB_1_R-dependent plasticity during critical developmental windows of circuit maturation is likely to have massively negative implications. In our experiments, we did not use cannabinoid receptor antagonists to block LTD, as the initial primary aim of our field recordings was to broadly characterize the effects of THC exposure in utero. As the initial primary aim of our field recordings was to broadly characterize the effects of THC exposure in utero, GABAergic antagonists to isolate NMDA-dependent LTP/LTD were not used. However, the increases in sIPSC frequency and decreased CB_1_ colocalization with VGAT likely decreased basal glutamatergic synaptic transmission, as our data show. Consistent with this model, prior work in other paradigms has shown that reducing GABAergic inhibition can restore synaptic plasticity when inhibitory neurotransmission is excessive (82), suggesting that targeting inhibitory signaling could partially normalize plasticity deficits following prenatal THC exposure.

In conclusion, the behavioral outcomes observed in this study demonstrate that prenatal THC exposure produces lasting impairments in hippocampal-dependent learning and memory during adolescence. These learning and memory deficits likely result from alterations in metaplastic balance at the circuit level. At the cellular level, prenatal THC-exposed rodents exhibited altered presynaptic release mechanisms leading to reduced AMPAR-mediated signaling. In addition, we observed increased inhibitory drive, which contributed to a shift in the E/I balance toward inhibition. At the molecular level, region-specific reorganization of CB_1_R expression, including reduced co-localization with VGAT-positive terminals, further suggests weakened endocannabinoid regulation of GABAergic transmission. Together, the tiered behavioral, electrophysiological, and molecular findings indicate that prenatal THC exposure induces long-lasting alterations in hippocampal circuitry that persist into adolescence. This data underscores the importance of continued investigation into the long-term, sex-specific, and developmental consequences of prenatal cannabis use, particularly given rising THC potency and increasing rates of use during pregnancy. This study, for the first time to our knowledge, addresses the intricate role of E/I balance in THC-mediated altered synaptic synchronicity, as well as how that is capable of altering metaplastic balance between LTP *versus* LTD

## Experimental procedures

### Animals

Timed-pregnant Sprague-Dawley rats were purchased from Envigo Laboratories. Pregnant rats received a daily dose of delta-9-tetrahydrocannabinol (THC; 5 mg/kg) orally administered through a buccopharyngeal cannula from gestational day (GD) 5 to PND 9. As previously described, delta-9 THC (Cayman) was dissolved in sesame oil (vehicle) (46). Control pregnant rats received the same volume (0.2 ml) of vehicle. Pups were assigned a unique identifier, and experimenters were blinded to treatment during the experiment. Experimental procedures were performed between PND 40 and 65 in offspring. Animals were housed in a vivarium on a 12 h:12 h light: dark cycle. All procedures were carried out in accordance with NIH guidelines and approved by the Auburn University Animal Care and Use Committee (IACUC). Sample size calculation, study design, statistical analysis, result reporting, and so on, followed ARRIVE guidelines.

### Behavioral experiments

#### Open field

To assess the general locomotor ability of rats, the open field test was performed by placing male rats in a transparent 60 × 60 cm plexiglass arena that they were allowed to explore for 10 min. Activity measures included mean speed (cm/s) and distance traveled (cm) in the arena. A virtual center square comprising half the arena was designated the “center zone,” and time spent in the center zone was compared to assess anxiety-like behavior (33, 34). The trial was recorded *via* a camera, and EthoVision XT 11.5 software was used to track the rat.

#### Novel arm y-maze

The Y-maze was used to assess working memory and spatial memory functions in rats, as described previously (83). The apparatus for the Y-maze test consisted of three plastic arms (7.5 cm wide × 38 cm long × 15 cm high) arranged at 120° to each other. Landmarks (geometric figures) were placed 5 cm above the available arms. The training sessions lasted 15 min, during which the animals could explore only two arms: the arm where they were placed (the start arm) and one of the other two arms (the familiar arm), located to the left or right of the entry arm. The third arm (the novel arm) was occluded by an opaque divider. The identity of the start, familiar, and novel arm was counterbalanced within groups. Animals were then returned to their home cages for 3 h. After this time, the animals were returned to the maze, with all arms open for this session. They were allowed to explore the maze for 10 min and then returned to their home cages. The distance traveled, and entries into the start and familiar arms were assessed during training to ensure no biases. The percentage of entries in the novel arm was compared during testing. The trials were recorded *via* a camera, and EthoVision XT 11.5 software was used to track the rat.

#### Morris water maze

The MWM was performed as previously described (9, 84). For hidden platform training, male rats were released from a semirandom starting location and was allowed 60 s to locate the platform, where the rat remained for 15 s. Rats underwent four training trials per day for 4 days. A probe trial was conducted 24 h and 1 week after the last hidden platform training trial. Path length to the hidden platform was assessed for hidden training trials, as this measure is generally considered immune to differences in swimming speed (85). For probe trials, the percent time in the target quadrant and platform-crossing index was examined.

### Electrophysiological recording

#### Preparations of acute hippocampal slices

Transverse or coronal hippocampal slices (350–400 μm) from male pups were prepared following euthanasia for all electrophysiological experiments. Using guidelines from the American Veterinary Medical Association (AVMA) 2013 edition, animals were euthanized. Animals were placed in the euthanasia chamber one at a time, followed by the addition of CO_2_ from a compressed gas cylinder at a low flow rate. Animal death was verified before removing from the chamber. For field recording experiments, rats were euthanized with isoflurane overdose. On confirmation of cessation of breathing, brains from rats were sliced using ice cold cutting solution (mM): 85 NaCl, 2.5 KCl, 4.0 MgSO_4_, 0.5 CaCl_2_, 1.25 NaH_2_PO_4_, 25 NaHCO_3_, 25 glucose, 75 sucrose, and 0.5 ascorbate saturated with 95% O_2_/5% CO_2_ following published methods (86). All slices were incubated at room temperature (RT) in artificial cerebrospinal fluid (aCSF) made of (mM) 124 NaCl, 24 NaHCO_3_, 10 glucose, 3 KCl, 1.5 MgSO_4_, 2.4 CaCl_2_, and 1.25 NaH_2_PO_4_ saturated with 95%O_2_/5% CO_2_ for at least 1 h before use (87). The recording aCSF solution was the same as the incubating solution. Recording electrodes pulled from thin-walled glass (TW150F-4, World Precision Instruments) had resistances of 2 to 3 MW and filled with the recording aCSF as internal solution containing (86, 88). The recordings were made using WinLTP software following published methods (35).

For patch clamp experiments, rats were euthanized by isoflurane overdose. On confirmation of cessation of breathing, rats were transcardially perfused following published methods using chilled sucrose aCSF containing (in mM) 86 NaCl, 75 sucrose, 2.5 KCl, 1.25 NaH_2_PO_4_, 25 NaHCO_3_, 25 glucose, 0.5 CaCl_2_, and 7 MgSO_4_ saturated with 95% O_2_/5% CO_2_ (86). Following decapitation, brains were removed, and hippocampal slices were sectioned and submerged in ice-cold sucrose aCSF. Slices were then transferred to holding aCSF containing (in mM) 125 NaCl, 2.5 KCl, 1.25 NaH_2_PO_4_, 25 NaHCO_3_, 20 glucose, 1 Na ascorbate, 3 Na pyruvate, 2 CaCl_2_, and 2 MgSO_4_ and incubated at 32 to 34 °C for 30 min. After heated recovery, slices were incubated at-3-34 °C for 30 min. After heated recovery, slices were incubated at RT for at least 1 h before recording. The recording aCSF solution contained (in mM) 125 NaCl, 2.5 KCl, 1.25 NaH_2_PO_4_, 25 NaHCO_3_, 20 glucose, 2.5 CaCl_2_, and 1.3 MgSO_4_ saturated with 95%O2/5% CO2. All solutions were filtered through a 0.2 mm filter immediately before use. Recording electrodes were pulled from thin-walled borosilicate glass (TW150F-4, World Precision Instruments) with resistances of 3 to 5 MW. For voltage-clamp recordings, electrodes were filled with a Cs-based internal solution containing (in mM) 135 Cs-gluconate, 5 CsCl, 0.5 EGTA, 10 Hepes, 2 MgCl2, 2 Na2-ATP, 0.3 Na2-GTP, 1 phosphocreatine, 1 QX-314 (Cl), 5 BAPTA, pH 7.2 to 7.3 (86, 88). For current-clamp recordings, electrodes were filled with a K-based internal solution containing (in mM) 142 K-gluconate, 4 KCl, 0.5 EGTA, 10 Hepes, 2 MgCl2, 2 Na2-ATP, 0.3 Na2-GTP, and 1 phosphocreatine. Internal solutions were diluted by £ 5% v/v with deionized water to an osmolality of ∼290 mOsm and then filtered using a 0.2 mm syringe-fitted nylon filter. Recordings were made using an Axopatch 200B amplifier and Digidata 1440B digitizer (Molecular Devices,). Analog signals were filtered at 2 kHz using an eight-pole Bessel filter (3 dB; Frequency Devices).

### Extracellular field recordings

Slices were transferred to a recording chamber submerged in aCSF bubbled with 95% O_2_/5% CO_2_ at 30 °C after at least 1 h of incubation. Field excitatory postsynaptic potentials (fEPSPs) were recorded from the hippocampal Schaffer collateral pathway. CA3 region was stimulated with a bipolar electrode, and the recording electrode was placed in CA1. fEPSPs were recorded in response to increasing stimulus intensity. Basal synaptic transmission was measured as the slope of fEPSPs. For paired-pulse ratio and LTP experiments, the current intensity was set to elicit fEPSPs with a slope equal to 50% of the maximum fEPSP. Interpulse intervals were set to 20, 50, 100, 150, and 200 ms in paired-pulse ratio experiments. In LTP experiments, after 10 min of stable baseline recording, induction was initiated by using theta-burst stimulation (TBS) (89). An inter-TBS interval of 20 s was applied to five TBS sweeps. For LTD recording, induction was given using two low-frequency stimulations (LFSs: 900 pulses at 1 Hz) delivered at 10 min intervals, preceded by 10 min of stable baseline. Stimulation intensity was set at 60% (during LFS) or 40% (all other times excluding LFS) of the maximum amplitude. LTP and LTD were measured as an average of fEPSP slopes from 50 to 60 min post induction. Field potentials were recorded using LTP 2.0 software with Axoclamp 2B and analyzed using WinLTP 2.0 software (40).

### Whole cell patch clamp

Whole-cell voltage-clamp recordings were obtained from pyramidal neurons in transverse/coronal brain slices. Between breaking into whole-cell configuration to establish a stable patch and starting recording, neurons were allowed to equilibrate *via* diffusion of the pipette solution for ≥5 min for spontaneous activity and ≥10 min for evoked experiments. AMPAR sEPSCs were recorded at −60 mV (90). To avoid the influence of recording manipulations on AMPAR sEPSCs at baseline, AMPAR activity was recorded prior to depolarized voltage-clamping. All spontaneous activity was recorded prior to stimulation for evoked recordings. Given the area of interest in the hippocampus and the age of experimental animals, AMPA receptors are assumed to be the principal population of glutamate receptor subtypes present besides NMDA receptors. We measured the frequency and amplitudes of sEPSCs to understand how spontaneous events are affected in our rodents. To measure changes in E/I balance, experiments were performed using similar slice preparation and recording protocols. While measuring E/I balance, sIPSCs, and sEPSCs were simultaneously recorded at −40 mV. This voltage was chosen as it captures both excitatory and inhibitory components of spontaneous synaptic currents. The frequency and amplitudes of simultaneously occurring sEPSCs and sIPSCs were subsequently compared (30). All sEPSC/sIPSC recordings were performed using a Cs-based internal solution to prevent cellular depolarization at holding potentials above threshold. For current-clamp recordings using a K-based internal solution, the holding current was first set to 0 pA to measure resting membrane potential, and bias current was applied to each cell to match baseline membrane potential at −70 mV prior to current stepping. Current steps were then applied from ±500 pA in 25 pA increments to measure input resistance, threshold potential, rheobase current, and spike frequency. For whole-cell evoked eE/IPSC recordings, a bipolar stimulus electrode (Microprobes for Life Science Model PI2ST32.0A3) was placed approximately 300 to 400 μm away from the patched cell in the stratum radiatum toward CA3. Brief current pulses (0.1 ms) were generated (AM Systems Model 2100) to elicit compound eE/IPSC events, and stepwise increases in stimulation intensity were used to establish maximum values for each component of the compound trace. Stimulation intensities and event amplitudes were normalized to percentages for each component, and sigmoidal stimulation-response data were subsequently fitted using nonlinear regression to calculate ES50 values (30).

### *In vivo* glutamate measurement (enzyme-coated microelectrode array)

Enzyme-immobilized amperometric microelectrode arrays (MEAs) were used to quantify KCl-evoked glutamate release in hippocampal subregions, DG, CA3, and CA1, of anesthetized rats, as we have previously described (91, 92, 93, 94). Ceramic multisite MEAs with eight paired platinum recording sites were purchased to measure glutamate and remove interferents (Quanteon, L.L.C). Sentinel sites were prepared with an inactive protein matrix consisting of bovine serum albumin (BSA) and glutaraldehyde and coated on the first pair of recording sites to block large molecule interferents. Next, a glutamate oxidase (GluOx) matrix was coated on the first pair of recording sites to oxidize glutamate into alpha-ketoglutarate and hydrogen peroxide, the reporter molecule. A size-exclusion layer was then electropolymerized onto the electrode to block large molecule interferents. Before implantation, the electrodes were calibrated, and sentinel sites were subtracted from the current obtained on the glutamate oxidase recording sites, producing a selective measure of extracellular glutamate. A glass micropipette (A-M systems) was pulled using a Narishige PC-10 and bumped to have an internal diameter of 10 to 15 μm and affixed to the microelectrode with the tip 75 to 100 μm from the reporter sites. The micropipette was then attached to a Picospritzer III (Parker Hannifin) and set to deliver consistent volumes of 100 nl of sterile-filtered isotonic 70 mM KCl. Rats were anesthetized with 4% isoflurane in an induction chamber and remained on isoflurane ventilation (1–2.5%). An infrared heating pad and rectal probe were used to maintain the body temperature at ∼37 °C to prevent anesthesia-induced hypothermia. When administered under controlled body temperature, isoflurane anesthesia does not alter resting glutamate concentrations (95). MEA recordings were taken in the CA1, CA3, and DG of the hippocampus, and stereotaxic coordinates were determined from bregma (96) [CA1 (ML: ± 3.2, AP: −3.7, DV: −3.4), CA3 (ML: ± 3.2, AP: −3.7, DV: −4.0), DG (ML: ± 3.2, AP: −2.1, DV: −4.1)]. MEA recordings were performed at a sampling rate of 10 Hz using constant potential amperometry (FAST-16 MkII). After reaching a stable baseline (20–45 min), the average of up to ten injections of 100 nl of 70 mM KCl spaced 2 to 3 min apart were compared per group. Data from some hippocampal subregions were excluded for reasons including clogging or failure of the MEA and death during surgery. Amperometric data were analyzed using a MATLAB program available for downloading at https://fastanalysis.wordpress.com/fast-analysis/. Details regarding calculations can be found in (97).

### Immunohistochemistry

Pups were perfused transcardially with ice-cold phosphate buffer (PB) following isoflurane overdose. Brains were harvested and immersion fixed in 4% paraformaldehyde in PBS at 4 °C for a minimum of 24 h. The samples were then transferred to a 15-mL Falcon tube containing a 30% sucrose solution in PBS and stored at 4 °C for a minimum of 24 h, or until the brains sank to the bottom of the tube (maximum 36 h). Excess moisture was removed from the tissue exterior, and tissue was flash-frozen in liquid nitrogen and stored at −80 °C. Tissues were then mounted in a Leica CM3050S cryostat using optimal cutting temperature compound (OCT, Cat# 4583, Sakura TissueTek), and 40-μm sagittal hippocampal slices were obtained and placed in 1M phosphate-buffered saline (PBS). An antigen retrieval protocol using citric acid buffer (anhydrous citric acid VWR), 0.2% Tween-20) was applied to minimize protein cross-linking during fixation. To improve the signal-to-noise ratio and reduce background staining, sections were treated with Sudan Black B (Sigma-Aldrich) for 30 min, and the solution was removed with 10 s of 70% ethanol, followed by 10 min in PBS. Sample sections were blocked and permeabilized in 5% donkey serum containing 0.5% Triton X-100 for 1 h at RT. Sections were incubated overnight with the primary antibodies CB_1_ and VGAT (Table 1). Sections were washed in PBS 3× for 10 min each and incubated with appropriate fluorophore-conjugated secondary antibodies for 2 h in the dark. After the secondary antibodies were washed, the slices were mounted with VECTASHIELD Antifade Mounting Media (Vector Laboratories, H-1900-2). Blocking and antibody incubation steps were performed on a shaker at 40 rpm at RT, while washes were performed at intervals of 55 rpm.

**Table 1:** List of antibodies

Antibodies	Host and type	Specificity	Source	Catalog #	RRID	Dilution
Primary antibodies						
GluA1	Mouse, monoclonal	M R	Invitrogen	MA5-18117	AB_2539491	1:1000
GluN2A	Rabbit, monoclonal	M R	Cell Signaling Technology	4205	AB_2112295	1:1000
GluN2B	Mouse, monoclonal	H M R	Abcam	ab28373	AB_776810	1:1000
VGLUT-1	Rabbit, monoclonal	H M R	Cell Signaling Technology	47181	AB_2797887	1:1000
Synaptophysin	Mouse, monoclonal	B R A F M H	EMD Millipore	MAB5258	AB_2313839	1:5000
PSD95	Rabbit, Monoclonal	H M R	Cell Signaling Technology	3409	AB_1264242	1:5000
CB1	Rabbit, monoclonal	H M R	Abcam	ab259323	AB_367620	1:200
VGAT	Mouse, monoclonal	H M R GP M	Synaptic Systems	131 011	AB_887872	1:500
Secondary antibody						
Anti-rabbit IgG Alexa Fluor 680	Goat	R	Invitrogen	A-21076	AB_2535736	1:5000
Anti-mouse IgG IRDye 800CW	Goat	M	Li-Cor	926-32210	AB_621842	1:5000
Anti-mouse IgG (H + L), Alexa Fluor 488	Donkey	M	Invitrogen	A10040	AB_2534016	1:1000
Anti-rabbit IgG (H + L) Alexa Fluo Plus 547	Donkey	R	Invitrogen	A21202	AB_2535790	1:1000

Fluorescence imaging was performed with a Nikon A1+ Confocal Scanning Laser Microscope with a 40× oil immersion lens. The laser power and gain settings were determined based on vehicle samples and were not adjusted subsequently. They were collected at a spatial sampling of 0.31 μm/pixel with a z-step of 2.12 μm. Images were analyzed using ImageJ (NIH), by delineating appropriate subregions and splitting the channels. Masks were created to identify individual pixels with the intensity of interest (CB_1_ and VGAT) in each channel. Furthermore, three distinct regions of interest (ROI 1–3) were identified in each hippocampal region (CA1, CA3, and DG). Individual pixel intensities were then integrated using custom-written MATLAB scripts (R2024b). The ratio or absolute value of intensities was quantified pixel-by-pixel within each region of interest.

### Immunoblotting

Hippocampal tissue from pups was homogenized in ice-cold lysis buffer (50 mM Tris–HCl pH 7.4, 150 mM NaCl, 0.5% Triton X-100, 1 mM EDTA, 3% sodium dodecyl sulfate, and 1% Na deoxycholate) with Roche protease inhibitor tablets (Pierce Protease inhibitor tablets, EDTA-Free, Thermo Fisher Scientific, A32965). Lysates were sonicated, incubated on ice for 30 min and centrifuged at 12,000*g* for 20 min at 4 °C. Supernatants were collected, and protein concentration was measured using BCA assay kit (Pierce BCA Protein Assay Kit, Thermo Fisher Scientific), with BSA as a standard. Samples were diluted in 4x reduced Laemmli buffer (Bio-Rad), boiled for 5 min at 95 °C, or heated for 5 min at 70 °C, and 20 to 40 μg of protein were resolved in 8% or 10% SDS-PAGE gels, and transferred onto 0.45 um Immun-Blot Low Fluorescence PVDF membranes (Bio-rad, Hercules). Transfer efficiency and total protein loading were confirmed by Ponceau S staining (Sigma-Aldrich, P7170). Membranes were blocked in 5% nonfat dry milk in Tris-buffered saline for 1 h at RT and probed with primary antibodies (Table 1), diluted in 5% BSA in tris-buffered saline with 0.1% Tween 20 (TBST) overnight at 4 °C. After washing three times with TBST, membranes were incubated with anti-rabbit/mouse secondary antibodies conjugated to IR680/IR800 diluted in Li-Cor (Li-Cor, 927-70001) for 1 h at RT. Secondary antibodies were washed with TBST, and membranes were imaged with the Azure 600 imaging system (Azure Biosystems, AZI600-01). Band intensities were quantified using ImageJ (NIH), using identical regions of interest for all lanes within the blots. All blots were images within the linear detection range and densitometry was performed only in nonsaturated exposures. For each sample, the raw intensity of the protein of interest was normalized to the corresponding Ponceau S signal from the same lane to correct for differences in protein loading and transfer efficiency. These Ponceau-normalized values were then normalized to the mean value of the Vehicle group on the same blot to enable comparison across treatment conditions while preserving within-blot variability.

Details of antibodies and dilutions used for IHC or immunoblotting are included in Table 1. All antibodies were commercially sourced and vendor-validated for specificity using standard methods such as knockout lines, secondary antibody comparison, or immunoprecipitation-mass spectrometry.

### Statistical analysis

Data analysis was performed using Clampfit11.0 and GraphPad Prism 8.0. Unless otherwise indicated, for bar graphs with two groups, statistical analysis consisted of unpaired *t*-tests, Welch’s *t* test if variances between the groups differed, or the Kolmogorov-Smirnov (KS) test if data did not pass the Shapiro-Wilk normality test. Repeated measures ANOVAs were used for line graphs unless there were missing values, in which case a mixed-effects model was used. In addition, the slopes for line graphs were obtained using linear regression (y = mx + b). Results were presented as mean ± standard deviation (SD), and differences between groups were considered statistically significant at *p* < 0.05. Wherever possible, graphs have been demonstrated with data points as a spread.

## Data availability

All data in the present study are either in the manuscript or or uploaded to the BioImage Archive DOI: https://doi.org/10.6019/S-BIAD3500.

## Institutional review board statement

The animal study protocol was approved by the Institutional Review Board of Auburn University (protocol code 2019-3572 approved on 8/14/2019).

## Conflict of interest

The authors declare that they have no conflicts of interest with the contents of this article.
